# Weight Misperception, Weight Dissatisfaction, and Weight Change Among a Swiss Population-Based Adult Sample

**DOI:** 10.3390/ijerph22081237

**Published:** 2025-08-08

**Authors:** Lucy Manca, Pedro Marques-Vidal

**Affiliations:** Lausanne University Hospital (CHUV), University of Lausanne, 1011 Lausanne, Switzerland

**Keywords:** weight dissatisfaction, weight misperception, weight gain, prospective study

## Abstract

Background/Objectives: We investigated the effect of weight misperception or dissatisfaction among individuals of normal BMI on their long-term weight changes. Methods: Data from the three follow-ups of the CoLaus|PsyCoLaus study (2009–2012, 2014–2017, and 2018–2021) conducted in Lausanne, Switzerland. Participants with a BMI < 25 kg/m^2^ were eligible. Weight misperception/dissatisfaction was assessed by questionnaires. Weight change over a 5- or 10-year period was categorized as stable (±5 kg), loss (<−5 kg), or gain (>+5 kg). Results: Overall, 1826 (66.1% women, 55.9 ± 9.9 years) and 1089 (62.7% women, 61.2 ± 9.6 years) participants of surveys 2009–2012 and 2014–2017, respectively, were retained for analysis. Prevalence of weight misperception was 15.9% (95% CI: 14.2–17.6) and 11.5% (95% CI: 9.6–13.5) in 2009–2012 and 2014–2017, respectively. The corresponding values for weight dissatisfaction were 28.9% (95% CI: 26.8–31.0) and 20.9% (95% CI: 18.6–23.5). After multivariable analysis, participants with weight misperception/dissatisfaction had a higher likelihood (*p* < 0.05) of presenting with weight loss: for weight misperception, odds ratio and 95% CI: 2.29 (1.30–4.02) and 2.66 (1.24–5.69) for 2009–2012 and 2014–2017, respectively; the corresponding values for weight dissatisfaction were 2.02 (1.22–3.35) and 2.40 (1.23–4.65). No associations between weight misperception/dissatisfaction with weight gain were found. Conclusions: Our study found that weight misperception/dissatisfaction in normal-weight, middle-aged individuals was associated with weight loss over time.

## 1. Introduction

Weight misperception, which can be defined as considering oneself as too heavy, despite a normative body mass index (BMI), is a frequent condition in the general population [1] and a powerful determinant of dietary intake. In a previous study, we have shown that women with a normal BMI who wished to lose weight reported lower energy and protein intake per kg of body weight, more signs of depression, and less satisfaction with their quality of life than those who did not wish to lose weight [2]. Whether this behavior succeeds in reducing or maintaining weight in the forthcoming years was not assessed. Indeed, contradictory effects of weight misperception have been reported, with one study showing an association with an increased incidence of obesity later in life [3], while two others showed a protective effect against weight gain, but mostly among people with overweight or obesity [4,5].

Weight dissatisfaction, which can be defined as a desire to lose weight despite a normal-weight, might lead to eating disorders [6], which in turn could promote weight gain [7,8]. Still, the available literature reports conflicting results between weight dissatisfaction and weight gain. Several studies reported that people dissatisfied with their body weight tend to increase their weight [3,9,10], while no such association was found in others [4,5,11].

Hence, given the contradictory findings regarding the effects of weight misperception and weight dissatisfaction on weight change, our objective was to assess the associations between weight misperception, weight dissatisfaction, and subsequent weight trajectories in a sample of normal-weight, middle-aged, community-dwelling subjects living in the city of Lausanne, Switzerland.

Our hypothesis was that, as reported in the literature, both weight misperception [3] and weight dissatisfaction [3,9,10] would lead to increased weight gain.

## 2. Materials and Methods

### 2.1. Study Population

Data from the first to the third follow-ups of the CoLaus|PsyCoLaus study was used. The aims and methodology of the CoLaus|PsyCoLaus study have been thoroughly described [12] and can be accessed at www.colaus-psycolaus.ch. Briefly, the CoLaus| PsyCoLaus study is a prospective cohort study assessing the association between cardiovascular and psychological illnesses and their risk factors. The cohort was randomly drawn from the city of Lausanne register of inhabitants aged 35 to 75. The baseline survey was conducted between 2003 and 2006 and included 6733 participants. The subsequent follow-up surveys were conducted in 2009–2012, 2014–2017, and 2018–2021. As no questions regarding weight were asked at the baseline, only data from the follow-up surveys was considered.

### 2.2. Ethical Statement

The institutional Ethics Committee of the University of Lausanne, which afterwards became the Ethics Commission of Canton Vaud (www.cer-vd.ch), approved the CoLaus|PsyCoLaus study (project number PB_2018-00038, reference 239/09). All participants gave their signed informed consent before entering the study.

### 2.3. Weight Misperception and Weight Dissatisfaction

Body weight and height were measured with participants barefoot and in light indoor clothes. Body weight was measured in kilograms to the nearest 100 g using a Seca® scale (Hamburg, Germany). Height was measured to the nearest 5 mm using a Seca® (Hamburg, Germany) height gauge. BMI was computed and categorized as normal if <25 kg/m^2^.

Participants were queried regarding their weight status using questionnaires regarding previous diagnosis by a doctor of excess weight, how they considered their own weight, and what they desired to do regarding their own weight (Appendix A). Participants were categorized as presenting with weight misperception if they considered their weight as excessive despite their normal BMI and as presenting with weight dissatisfaction if they desired to lose weight. Weight misperception thus relates to the self-evaluation of one’s body weight and can be considered as a status, while weight dissatisfaction is a proactive condition based on the desire to lose weight.

### 2.4. Weight Change

Five-year (for both first and second follow-ups) and 10-year weight changes (for the first follow-up) were assessed either continuously [13] or categorized into stable (±5 kg), loss (>5 kg decrease), and gain (>5 kg increase) as previously performed [14].

### 2.5. Covariates

Participants answered questionnaires regarding their socio-economic status, lifestyle, and drug treatment. Smoking was self-reported and categorized as never, former (irrespective of the time since quitting), and current. Marital status was categorized as living alone (i.e., single, divorced, or widowed) or living with a partner. Education was categorized as low (compulsory or apprenticeship), middle (high school), or high (university). Nationality was categorized as born/not born in Switzerland. Alcohol consumption was categorized as drinker and non-drinker.

Blood pressure (BP) was measured using an Omron® HEM-907 automated oscillometric sphygmomanometer (Omron, Kyoto, Japan) after at least a 10 min rest in a seated position, and the average of the last two measurements was used. Hypertension was defined by a systolic blood pressure (SBP) ≥ 140 mm Hg, a diastolic blood pressure (DBP) ≥ 90 mm Hg, or the presence of antihypertensive drug treatment.

Dietary intake was assessed using a validated, self-administered, semi-quantitative food frequency questionnaire (FFQ), which also included portion size [15,16]. Briefly, this FFQ assesses the dietary intake of the previous 4 weeks and consists of 97 different food items that account for more than 90% of the intake of calories, protein, fat, carbohydrates, alcohol, cholesterol, vitamin D, and retinol, as well as 85% of fiber, carotene, and iron. Each participant brought along their filled-in FFQ, which was checked for completion by trained interviewers the day of the visit. Total energy intake (TEI) was used as a covariate.

### 2.6. Eligibility, Inclusion, and Exclusion Criteria

Only participants with normal BMI (<25 kg/m^2^) were considered as eligible. Participants were excluded if they (1) did not respond to the weight questionnaire; (2) had no follow-up; and (3) missed any covariate.

### 2.7. Statistical Analysis

Statistical analysis was conducted using Stata version 18.0 for Windows (Stata corp., College Station, TX, USA). Descriptive results were expressed as number of participants (column percentage) for categorical variables and as average ± standard deviation or median [interquartile range] for continuous variables. For bivariate analyses, comparisons were performed using the chi-square exact test for categorical variables and the Student’s t-test or Kruskal–Wallis test for continuous variables. Between-group comparisons were performed using polytomous logistic regression for categorical variables and analysis of variance (ANOVA) for continuous variables. For multivariable analyses, results were expressed as relative risk ratio (95% confidence interval) for categorical variables and as adjusted mean ± standard error for continuous variables. Multivariable models were adjusted on gender, age (continuous), marital status, alcohol consumption, smoking status (never, former, current), total energy intake (continuous), and previous diagnoses of overweight, hypertension, and diabetes.

A sensitivity analysis was conducted using inverse probability weighting to account for exclusions. Briefly, the probability of being included was computed using a logistic model, including all the variables significantly different between included and excluded participants, and the inverse of this probability was used in the polytomous logistic regression models. An analysis stratifying by gender was also carried out.

We considered a two-tailed *p* < 0.05 to be statistically significant.

## 3. Results

### 3.1. Study Population

For the first study period of the initial 5064 participants, 2180 (43.1%) were considered eligible, of whom 337 (15.4%) were excluded due to the lack of follow-up, and a further 17 (0.8%) due to the lack of covariates (Figure S1, panel A). For the second study period of the initial 4881 participants, 1857 (38.0%) were considered eligible, of whom 414 (22.3%) were excluded due to the lack of follow-up and a further 354 (19.1%) due to the lack of covariates (Figure S1, panel B). The characteristics of the excluded and included participants according to study period are summarized in Table S1. Included participants were younger, more frequently women, with a higher educational level, and more frequently alcohol drinkers and never smokers; included participants also presented less frequently with hypertension and diabetes.

### 3.2. Weight Misperception: Characteristics of Participants and Effect on 5-Year Weight Changes

The results of the bivariate analysis of the characteristics of the participants according to presence or absence of weight misperception are provided in Table 1. At the first follow-up, there were 279 participants with weight misperception, corresponding to 16.1% (95% CI: 14.4–17.9) of the sample; at the second follow-up, this figure was 11.7% (95% CI: 9.8–13.9). On bivariate analysis, and in both study periods, participants with weight misperception were younger, more frequently women, with a higher BMI, waist circumference, and abdominal obesity, and reported more frequently having been told that they were overweight and had a lower TEI (Table 1).

The results of the bivariate and multivariable analysis of 5-year weight change according to presence or absence of weight misperception for both study periods are provided in Table 2 and the individual values of weight change in Figure S2. On bivariate analysis, no difference was found regarding average weight gain according to weight misperception status. When weight changes were categorized, participants with weight misperception presented more frequently with changes over 5 kg (both loss and gain, Table 2). After multivariable analysis, participants with weight misperception had a higher likelihood of presenting with weight loss > 5 kg, while no differences were found for weight gain, and similar findings were obtained after inverse probability weighting (Table 2) or after stratifying on gender (Table S2).

### 3.3. Weight Dissatisfaction: Characteristics of Participants and Effect on 5-Year Weight Changes

At the first follow-up, there were 505 participants with weight dissatisfaction, corresponding to 29.2% (95% CI: 27.0–31.4) of the sample; at the second follow-up, this figure was 21.3% (95% CI: 18.8–23.9). The results of the bivariate analyses of the characteristics of participants according to presence or absence of weight dissatisfaction are presented in Table 3. In both study periods, participants with weight dissatisfaction were more often women, younger, of a higher educational level, had a higher BMI and waist circumference, presented more often with abdominal obesity, and had a lower TEI. Participants with weight dissatisfaction were more likely to have been diagnosed as overweight and presented less frequently with hypertension or diabetes.

The results of the bivariate and multivariable analyses of 5-year weight change according to the presence or absence of weight dissatisfaction are presented in Table 4 and the individual values of weight change in Figure S3. On bivariate analysis, no significant difference was observed in absolute weight change. However, when weight changes were categorized, participants with weight dissatisfaction had significantly higher weight changes–whether it was gain or loss. After multivariable analysis, participants with weight dissatisfaction had a higher likelihood of presenting with weight loss > 5 kg, while no differences were found for weight gain, and similar findings were obtained after inverse probability weighting (Table 4) or stratifying on gender (Table S3).

### 3.4. Weight Misperception or Dissatisfaction: Effect on 10-Year Weight Changes

Of the initial 5064 participants, 2180 (43.1%) were considered as eligible, of whom 625 (28.8%) were excluded due to the lack of follow-up and a further 14 (0.6%) due to the lack of covariates (Figure S1, panel C). The characteristics of the participants according to the presence of weight misperception or weight dissatisfaction are summarized in Table S4. Participants with weight misperception or weight dissatisfaction were more often women, younger, had a higher BMI and waist circumference, presented more often with abdominal obesity, and were more likely to have been diagnosed as overweight.

The results of the bivariate and multivariable analyses of 10-year weight change according to the presence or absence of weight misperception or weight dissatisfaction are presented in Table S5. On bivariate analysis, participants with weight misperception had a higher weight increase, while no significant difference was observed for participants with weight dissatisfaction. When weight changes were categorized, participants with weight misperception or dissatisfaction had significantly higher weight changes—whether it was gain or loss. After multivariable analysis, participants with weight misperception had a higher likelihood of presenting either with weight loss > 5 kg or weight gain > 5 kg; participants with weight dissatisfaction had a higher likelihood of presenting with weight loss > 5 kg, but no differences were found for weight gain (Table S5).

## 4. Discussion

In this study, participants with weight misperception or with weight dissatisfaction had a higher likelihood of presenting with weight loss > 5 kg after five or ten years of follow-up, while no associations were found for weight gain.

### 4.1. Weight Misperception or Dissatisfaction: Effect on Weight

Among obese individuals, the prevailing notion is that misperceiving oneself as a normal weight is an obstacle to weight loss and should be ‘corrected’. However, studies have found not only that this notion is incorrect but also that it produces the opposite effect. Correctly identifying oneself as overweight—in the case of people who are obese—induces weight gain over time [9,17]. The hypotheses are that this effect may be the result of multiple weight loss attempts [8] or of stress-induced overeating [7].

Contrary to our initial hypothesis, no weight gain was found among participants with weight misperception or with weight dissatisfaction relative to participants devoid of those conditions. Our results are partly in agreement with those of Sonneville et al. [5] as well as Rancourt et al. [4], which showed that weight misperception is associated with lower weight gain. Still, both studies focused on individuals who were obese or at least overweight and evaluated the effect of misperceiving themselves as being of normal-weight—which they found was protective against future weight gain.

Conversely, our results contradict those of Robinson et al. [7,8], Sutin & Terracciano [3], Haynes et al. [17], Feng & Wilson [9], Laraia et al. [10], and Wang et al. [11], the majority of which showed that weight misperception and/or weight dissatisfaction were linked to future weight gain—only Wang et al. [11] showed no link. This contradiction between the results of these studies and our own might be due to the characteristics of the studied population, notably their age, gender, or BMI. For example, several of the previous studies focused mainly on people of a younger age [3,8,10], only on women [10], or included individuals with a high BMI [8,9,10,17]. Our participants could also be more health conscious and thus more frequently incorporate diet or increased physical activity into their lifestyles, consequently leading to weight loss. For instance, a study conducted in Spain showed that in older adults with metabolic syndrome, more ambitious desired weight loss goals were associated with improvements in diet and cardiovascular health [18]. It is therefore possible that weight dissatisfaction leads to stronger actions aimed at reducing weight, even in subjects with normal BMI. Indeed, participants with weight misperception or with weight dissatisfaction had lower TEI on bivariate analysis; however, this difference was absent after multivariable adjustment in the first but not in the second follow-up. Still, the literature reports contradictory effects of weight dissatisfaction on diet and lifestyle: a study in Switzerland found no differences regarding overall diet quality between women with or without weight dissatisfaction [19], while a similar study in Spain concluded that body dissatisfaction was associated with a low score of the Mediterranean diet [20], and another study in Saudi Arabia reported little difference in diet and lifestyle behaviors according to the presence or absence of weight dissatisfaction [21]. Finally, given the relatively old age of our participants, it is also possible that they present with a lower prevalence of eating disorders, as it has been suggested that the frequency of eating disorders decreases with age [22,23], although this statement has been challenged [24]. It would be of interest to explore this possibility in the future.

Overall, our results suggest that, in this sample of apparently healthy, community-dwelling people with normal BMI, both weight misperception and weight dissatisfaction are associated with future weight loss but not with weight gain.

### 4.2. Implications for Clinical Practice

Our findings suggest that, in middle-aged people with normal-weight, weight dissatisfaction or weight misperception is not associated with an increased risk of weight gain. Hence, it might not be necessary for health professionals to manage this dissatisfaction/misperception, unless there is strong evidence of pathological behavior. Nevertheless, it would be important that our findings be confirmed or disaffirmed in other studies focusing on middle-aged, apparently healthy, community-dwelling samples.

### 4.3. Strengths and Limitations

The strengths of this study are that it is of a relatively large size compared to others [10]. The follow-up period was also longer than previous studies [9,11]. This study was carried out in a sample of middle-aged, normal-weight, general population, while many studies focused on people of a younger age [3,8,10], or only on women [10], or included individuals with a high BMI [8,9,10,17]. Additionally, this study looked at weight misperception as well as weight dissatisfaction.

This study also has some limitations worth acknowledging. Firstly, it was conducted in a single location, so its generalizability in other settings must be verified. Moreover, we cannot exclude a selection bias, with only the most motivated and health-conscious people accepting a follow-up. Still, the results were similar after inverse probability weighting. Furthermore, weight misperception and weight dissatisfaction were assessed via simple questions, and the diagnosis might not be as precise as via a full interview. Finally, even though standardization of responses can be achieved by questionnaires, it might not capture the full subtleties and severity of self-perceptions, thus leading to over- or underestimation biases. Still, other studies used a similar methodology [3,4,5,8,9,11].

## 5. Conclusions

Our study found that weight misperception and weight dissatisfaction in normal-weight, middle-aged individuals were associated with weight loss over time. These findings suggest that addressing these elements in this population may not be as critical for preventing weight gain as previously thought and that clinicians do not need to intervene in all cases of misperception/dissatisfaction, particularly in older, normal-weight individuals, unless an eating disorder or distress is present.

## Figures and Tables

**Table 1:** Baseline characteristics of participants according to weight misperception, stratified by study period, CoLaus|PsyCoLaus study, Lausanne, Switzerland.

	Follow-Up 1 to 2			Follow-Up 2 to 3		
	No (n = 1452)	Yes (n = 279)	*p*-Value	No (n = 917)	Yes (n = 123)	*p*-Value
Women (%)	915 (63.0)	224 (80.3)	<0.001	551 (60.1)	100 (81.1)	<0.001
Age (years)	56.4 ± 10.1	53.3 ± 8.7	<0.001	61.7 ± 9.7	57.5 ± 8.7	<0.001
Born in Switzerland (%)	976 (67.2)	195 (69.9)	0.38	626 (68.3)	83 (68.0)	0.96
Educational level (%)			0.087			0.21
High	398 (27.4)	94 (33.7)		264 (28.8)	38 (31.1)	
Middle	423 (29.1)	79 (28.3)		272 (29.7)	43 (35.2)	
Low	631 (43.5)	106 (38.0)		381 (41.5)	41 (33.6)	
Living as a couple (%)	799 (55.0)	147 (52.7)	0.47	602 (65.6)	80 (65.6)	0.99
Body mass index (kg/m^2^)	22.1 ± 1.9	23.5 ± 1.2	<0.001	22.1 ± 1.9	23.7 ± 1.3	<0.001
Waist (cm)	81.2 ± 8.0	84.4 ± 7.4	<0.001	80.6 ± 8.3	82.6 ± 7.8	0.012
Abdominal obesity (%)	84 (5.8)	57 (20.4)	<0.001	38 (4.1)	14 (11.5)	<0.001
Overweight diagnosis (%)	47 (3.3)	37 (13.6)	<0.001	23 (2.5)	18 (15.1)	<0.001
Alcohol drinker (%)	1136 (78.2)	228 (81.7)	0.19	697 (80.0)	100 (87.0)	0.076
Smoking status (%)						0.78
Never	653 (45.0)	123 (44.1)	0.67	409 (44.6)	57 (46.7)	
Former	483 (33.3)	100 (35.8)		340 (37.1)	46 (37.7)	
Current	316 (21.8)	56 (20.1)		168 (18.3)	19 (15.6)	
Hypertension (%)	380 (26.2)	60 (21.5)	0.10	262 (28.6)	25 (20.5)	0.060
Diabetes (%)	38 (2.6)	2 (0.7)	§ 0.054	22 (2.4)	2 (1.6)	§ 0.60
Energy intake (kcal)	1660 (1329–2075)	1624 (1240–1982)	† 0.036	1660 [1324–2031]	1560 [1107–1878]	† 0.004

Results are expressed as number of participants (column percentage) for categorical variables and as average ± standard deviation or median [interquartile range] for continuous variables. Between-group comparisons using chi-square or Fisher’s exact test (§) for categorical variables and Student’s *t*-test or Kruskal–Wallis test (†) for continuous variables.

**Table 2:** Bivariate and multivariable analysis of the 5-year weight changes of participants according to weight misperception, stratified by study period, CoLaus|PsyCoLaus study, Lausanne, Switzerland.

	Follow-Up 1 to 2			Follow-Up 2 to 3		
	No (n = 1452)	Yes (n = 279)	*p*-Value	No (n = 917)	Yes (n = 123)	*p*-Value
**Bivariate**						
Weight change (kg)	0.7 ± 3.7	1.1 ± 4.4	0.103	0.1 ± 3.2	0.1 ± 4.1	0.873
Weight change (%)			<0.001			0.018
Loss > 5 kg	56 (3.9)	19 (6.8)		43 (4.7)	12 (9.8)	
Stable	1269 (87.4)	221 (79.2)		827 (90.2)	100 (82.0)	
Gain > 5 kg	127 (8.8)	39 (14.0)		47 (5.1)	10 (8.2)	
**Multivariable**						
Weight change (kg)	0.78 ± 0.10	0.74 ± 0.23	0.865	0.11 ± 0.12	−0.35 ± 0.33	0.196
Weight change						
Loss > 5 kg	-	2.20 (1.24–3.93)	0.007	-	3.15 (1.44–6.90)	0.004
Stable	-	1 (ref)		-	1 (ref)	
Gain > 5 kg	-	1.38 (0.90–2.10)	0.142	-	1.98 (0.89–4.40)	0.092
Weight change, weighted						
Loss > 5 kg	-	2.11 (1.15–3.88)	0.015	-	2.89 (1.22–6.86)	0.016
Stable	-	1 (ref)		-	1 (ref)	
Gain > 5 kg	-	1.38 (0.91–2.09)	0.127	-	2.18 (0.93–5.09)	0.072

For bivariate analyses, results are expressed as number of participants (column percentage) for categorical variables and as average ± standard deviation for continuous variables. Between-group comparisons using chi-square for categorical variables and Student’s t-test for continuous variables. For multivariable analyses, results are expressed as relative risk ratios (95% confidence interval) for categorical variables and as adjusted mean ± standard error for continuous variables. Between-group comparisons were performed using polytomous logistic regression for categorical variables and ANOVA for continuous variables. Both multivariable models were adjusted for gender, age (continuous), marital status, alcohol consumption, smoking status (never, former, current), and diagnoses of overweight, hypertension, and diabetes.

**Table 3:** Baseline characteristics of participants according to weight dissatisfaction, stratified by study period, CoLaus|PsyCoLaus study, Lausanne, Switzerland.

	Follow-Up 1 to 2			Follow-Up 2 to 3		
	No (n = 1226)	Yes (n = 505)	*p*-Value	No (n = 818)	Yes (n = 221)	*p*-Value
Women (%)	744 (60.7)	395 (78.2)	<0.001	479 (58.6)	171 (77.4)	<0.001
Age (years)	57.0 ± 10.1	53.0 ± 8.8	<0.001	62.2 ± 9.8	57.6 ± 8.2	<0.001
Born in Switzerland (%)	841 (68.6)	330 (65.3)	0.19	557 (68.1)	152 (68.8)	0.85
Educational level (%)			<0.001			0.018
High	317 (25.9)	175 (34.7)		224 (27.4)	78 (35.3)	
Middle	353 (28.8)	149 (29.5)		245 (30.0)	70 (31.7)	
Low	556 (45.4)	181 (35.8)		349 (42.7)	73 (33.0)	
Living as a couple (%)	686 (56.0)	260 (51.5)	0.090	545 (66.6)	137 (62.0)	0.20
Body mass index (kg/m^2^)	21.9 ± 1.9	23.2 ± 1.4	<0.001	22.0 ± 1.9	23.3 ± 1.4	<0.001
Waist (cm)	81.1 ± 8.1	83.2 ± 7.6	<0.001	80.5 ± 8.4	82.1 ± 7.6	0.012
Abdominal obesity (%)	68 (5.5)	73 (14.5)	<0.001	33 (4.0)	19 (8.6)	0.006
Overweight diagnosis (%)	40 (3.3)	44 (8.8)	<0.001	19 (2.4)	22 (10.1)	<0.001
Alcohol drinker (%)	949 (77.4)	415 (82.2)	0.027	625 (80.5)	172 (81.9)	0.66
Smoking status (%)			0.41			0.29
Never	553 (45.1)	223 (44.2)		376 (46.0)	90 (40.7)	
Former	402 (32.8)	181 (35.8)		301 (36.8)	85 (38.5)	
Current	271 (22.1)	101 (20.0)		141 (17.2)	46 (20.8)	
Hypertension (%)	332 (27.1)	108 (21.4)	0.013	237 (29.0)	50 (22.6)	0.060
Diabetes (%)	35 (2.9)	5 (1.0)	§ 0.020	21 (2.6)	3 (1.4)	§ 0.29
Energy intake (kcal)	1671 [1342–2076]	1611 [1256–2007]	† 0.011	1678 [1347–2043]	1527 [1140–1903]	† < 0.001

Results are expressed as number of participants (column percentage) for categorical variables and as average ± standard deviation or median [interquartile range] for continuous variables. Between-group comparisons using chi-square or Fisher’s exact test (§) for categorical variables and Student’s t-test or Kruskal–Wallis test (†) for continuous variables.

**Table 4:** Bivariate and multivariable analysis of the 5-year weight changes of participants according to weight dissatisfaction, stratified by study period, CoLaus|PsyCoLaus study, Lausanne, Switzerland.

	Follow-Up 1 to 2			Follow-Up 2 to 3		
	No (n = 1226)	Yes (n = 505)	*p*-Value	No (n = 818)	Yes (n = 221)	*p*-Value
**Bivariate**						
Weight change (kg)	0.8 ± 3.7	0.8 ± 4.0	0.988	0.1 ± 3.2	0.1 ± 3.7	0.871
Weight change (%)			0.023			0.038
Loss > 5 kg	46 (3.8)	29 (5.7)		37 (4.5)	18 (8.1)	
Stable	1073 (87.5)	417 (82.6)		740 (90.5)	187 (84.6)	
Gain > 5 kg	107 (8.7)	59 (11.7)		41 (5.0)	16 (7.2)	
** Multivariable **						
Weight change (kg)	0.91 ± 0.11	0.44 ± 0.17	0.021	0.18 ± 0.12	−0.40 ± 0.25	0.040
Weight change						
Loss > 5 kg	-	1.88 (1.12–3.17)	0.017	-	2.88 (1.45–5.74)	0.003
Stable	-	1 (ref)		-	1 (ref)	
Gain > 5 kg	-	1.07 (0.74–1.55)	0.706	-	1.37 (0.70–2.69)	0.363
Weight change, weighted						
Loss > 5 kg	-	1.80 (1.10–2.92)	0.018	-	2.77 (1.26–6.08)	0.011
Stable	-	1 (ref)		-	1 (ref)	
Gain > 5 kg	-	1.07 (0.74–1.54)	0.732	-	1.45 (0.73–2.91)	0.292

For bivariate analyses, results are expressed as number of participants (column percentage) for categorical variables and as average ± standard deviation for continuous variables. Between-group comparisons using chi-square for categorical variables and Student’s *t*-test for continuous variables. For multivariable analyses, results are expressed as relative risk ratios (95% confidence interval) for categorical variables and as adjusted mean ± standard error for continuous variables. Between-group comparisons were performed using polytomous logistic regression for categorical variables and ANOVA for continuous variables. Both multivariable models were adjusted for gender, age (continuous), marital status, alcohol consumption, smoking status (never, former, current), and diagnoses of overweight, hypertension, and diabetes.

## Data Availability

The data of the CoLaus|PsyCoLaus study used in this article cannot be fully shared, as they contain potentially sensitive personal information on participants. According to the Ethics Committee for Research of the Canton of Vaud, sharing these data would be a violation of the Swiss legislation with respect to privacy protection. However, coded individual-level data that do not allow researchers to identify participants are available upon request to researchers who meet the criteria for data sharing of the CoLaus|PsyCoLaus Datacenter (CHUV, Lausanne, Switzerland). Any researcher affiliated with a public or private research institution who complies with the CoLaus|PsyCoLaus standards can submit a research application to research.colaus@chuv.ch or research.psycolaus@chuv.ch. Proposals requiring baseline data only will be evaluated by the baseline (local) Scientific Committee (SC) of the CoLaus and PsyCoLaus studies. Proposals requiring follow-up data will be evaluated by the follow-up (multicentric) SC of the CoLaus|PsyCoLaus cohort study. Detailed instructions for gaining access to the CoLaus|PsyCoLaus data used in this study are available at www.colaus-psycolaus.ch/professionals/how-to-collaborate/.

## Supplementary Materials

The following supporting information can be downloaded at: https://www.mdpi.com/article/10.3390/ijerph22081237/s1, Figure S1: selection of the participants; Figure S2: individual 5- and 10-year weight changes among participants with weight misperception; Figure S3: individual 5- and 10-year weight changes among participants with weight dissatisfaction. Table S1: comparison of baseline characteristics between included and excluded participants, CoLaus|PsyCoLaus study, stratified by study period, Lausanne, Switzerland; Table S2: multivariable analysis of the 5-year weight changes of participants according to weight misperception, stratified by study period and sex, CoLaus|PsyCoLaus study, Lausanne, Switzerland; Table S3: multivariable analysis of the 5-year weight changes of participants according to weight dissatisfaction, stratified by study period and gender, CoLaus|PsyCoLaus study, Lausanne, Switzerland; Table S4: baseline characteristics of participants according to weight misperception or weight dissatisfaction for the 10-year study, CoLaus|PsyCoLaus study, Lausanne, Switzerland; Table S5: bivariate and multivariable analysis of the 10-year weight changes of participants according to weight misperception or weight dissatisfaction, CoLaus|PsyCoLaus study, Lausanne, Switzerland.

## Abbreviations

The following abbreviations are used in this manuscript:

ANOVA	Analysis of variance
BMI	Body mass index
BP	Blood pressure
CI	Confidence interval
FFQ	Food frequency questionnaire
TEI	Total energy intake
