# Supplementary material for: Weight Misperception, Weight Dissatisfaction, and Weight Change Among a Swiss Population-Based Adult Sample
Source: Int J Environ Res Public Health. 2025 Aug 8;22(8):1237. doi: 10.3390/ijerph22081237 (PMC12386205; doi:10.3390/ijerph22081237)
Supplement: Supplementary file 1 [file ijerph-22-01237-s001.zip › Figure S2.pptx]

## Slide 1
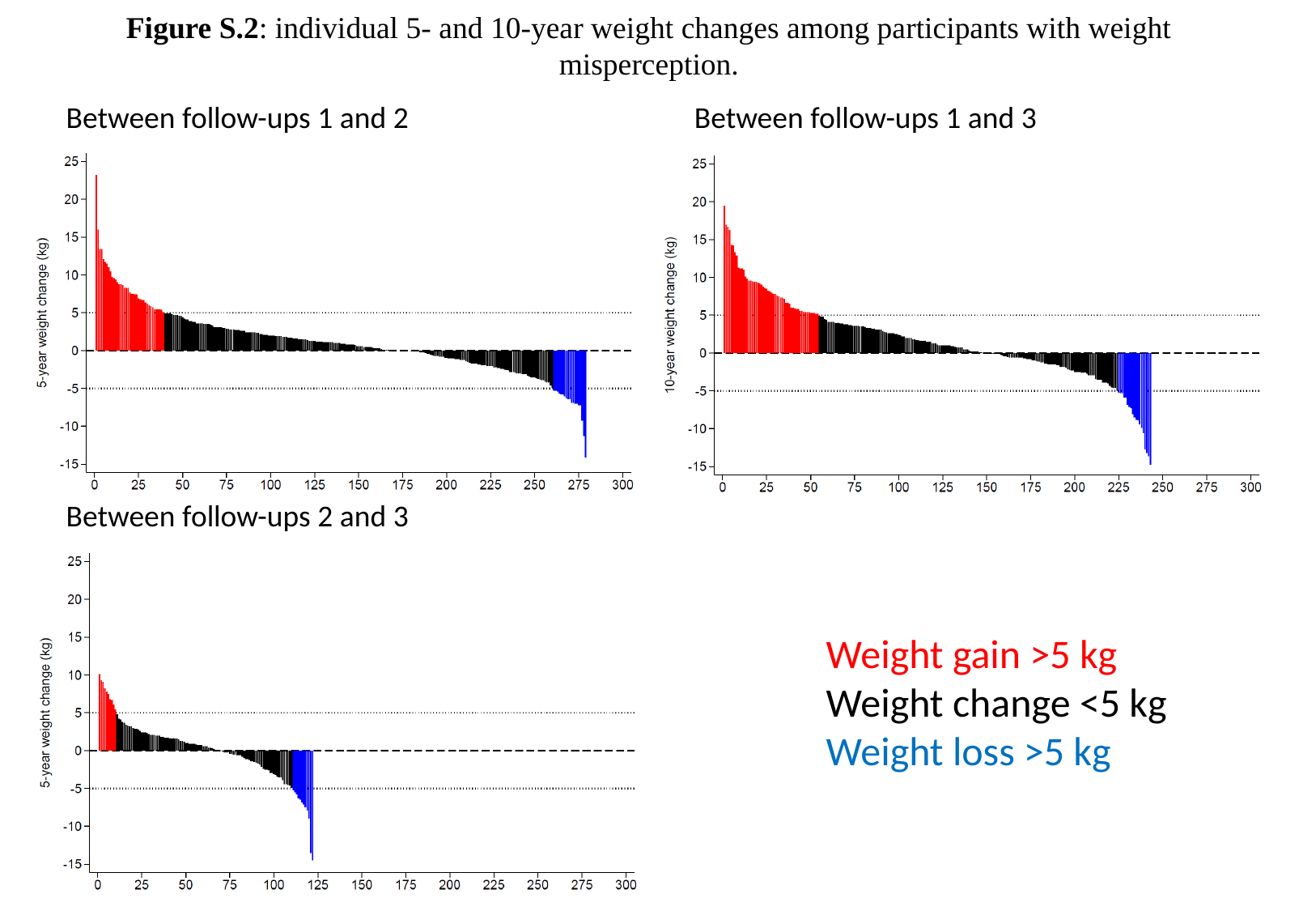

Figure S.2: individual 5- and 10-year weight changes among participants with weight misperception.
Between follow-ups 1 and 2
Between follow-ups 1 and 3
Between follow-ups 2 and 3
Weight gain >5 kg
Weight change <5 kg
Weight loss >5 kg
