# Supplementary material for: Weight Misperception, Weight Dissatisfaction, and Weight Change Among a Swiss Population-Based Adult Sample
Source: Int J Environ Res Public Health. 2025 Aug 8;22(8):1237. doi: 10.3390/ijerph22081237 (PMC12386205; doi:10.3390/ijerph22081237)
Supplement: Supplementary file 1 [file ijerph-22-01237-s001.zip › Supplementary tables.pdf]

SUPPLEMENTARY INFORMATION

Table S1 : comparison of baseline characteristics between included and excluded participants, CoLaus|PsyCoLaus study, stratified by study period, Lausanne, Switzerland.

	Follow-up 1 to 2			Follow-up 2 to 3		
	**Included (n=1731)**	**Excluded (N=449)**	**p-value**	**Included (n=1039)**	**Excluded (N=818)**	**p-value**
Women (%)	1139 (65.8)	276 (61.5)	0.087	650 (62.6)	565 (69.1)	0.003
Age (years)	55.9 ± 9.9	57.6 ± 10.8	0.001	61.2 ± 9.7	62.4 ± 11.1	0.011
Born in Switzerland (%)	1,171 (67.6)	281 (62.6)	0.043	709 (68.2)	455 (67.3)	0.69
Educational level (%)			<0.001			0.060
High	492 (28.4)	86 (19.2)		302 (29.1)	173 (25.6)	
Middle	502 (29.0)	107 (23.8)		315 (30.3)	189 (28.0)	
Low	737 (42.6)	256 (57.0)		422 (40.6)	313 (46.4)	
Living in couple (%)	946 (54.7)	212 (47.2)	0.005	682 (65.6)	269 (56.9)	0.001
Body mass index (kg/m²)	22.3 ± 1.9	22.3 ± 1.9	0.91	22.3 ± 1.9	22.2 ± 2.0	0.062
Waist (cm)	81.7 ± 8.0	82.6 ± 8.1	0.034	80.9 ± 8.2)	81.1 ± 8.5	0.61
Abdominal obesity (%)	141 (8.1)	47 (10.5)	0.12	52 (5.0)	49 (6.0)	0.34
Overweight diagnosis (%)	84 (4.9)	22 (5.2)	0.79	41 (4.0)	30 (4.1)	0.92
Alcohol drinker (%)	1,364 (78.8)	310 (69.0)	<0.001	797 (80.8)	487 (70.7)	<0.001
Smoking status (%)			<0.001			<0.001
Never	776 (44.8)	144 (33.6)		466 (44.9)	294 (40.4)	
Former	583 (33.7)	118 (27.6)		386 (37.2)	244 (33.6)	
Current	372 (21.5)	166 (38.8)		187 (18.0)	189 (26.0)	
Hypertension (%)	440 (25.4)	146 (32.6)	0.002	287 (27.6)	294 (36.0)	<0.001
Diabetes (%)	40 (2.3)	31 (6.9)	<0.001	24 (2.3)	28 (3.5)	0.14
Energy intake (kcal)	1654 [1316-2058]	1610 [1213-2047]	§ 0.050	1651 [1313-2011}	1541 [1182-1961]	§ <0.001

Results are expressed as number of participants (column percentage) for categorical variables and as average ± standard deviation or median [interquartile range] for continuous variables. Between-group comparisons using chi-square for categorical variables and student's t-test or Kruskal-Wallis test (§) for continuous variables.

Table S2: multivariable analysis of the 5-year weight changes of participants according to weight misperception, stratified by study period and sex, CoLaus|PsyCoLaus study, Lausanne, Switzerland.

	Follow-up 1 to 2			Follow-up 2 to 3		
	**No**	**Yes**	**p-value**	**No**	**Yes**	**p-value**
**Misperception, men**						
Weight change (kg)	0.92 ± 0.16	-0.36 ± 0.52	0.019	0.09 ± 0.17	-1.38 ± 0.70	0.045
Weight change						
Loss >5 kg	-	4.45 (1.42 - 13.9)	0.010	-	11.3 (3.11 - 41.2)	<0.001
Stable	-	1 (ref)		-	1 (ref)	
Gain >5 kg	-	0.56 (0.17 - 1.91)	0.356	-	3.25 (0.72 - 14.72)	0.126
**Misperception, women**						
Weight change (kg)	0.71 ± 0.12	0.99 ± 0.26	0.331	0.12 ± 0.15	-0.06 ± 0.38	0.669
Weight change						
Loss >5 kg	-	1.82 (0.93 - 3.58)	0.081	-	1.89 (0.66 - 5.39)	0.236
Stable	-	1 (ref)		-	1 (ref)	
Gain >5 kg	-	1.69 (1.05 - 2.71)	0.030	-	1.62 (0.62 - 4.21)	0.326

Results are expressed as relative risk ratio and (95% confidence interval) for categorical variables and as adjusted mean ± standard error for continuous variables. Between-group comparisons performed using polytomous logistic regression for categorical variables and ANOVA for continuous variables. Both multivariable models were adjusted on age (continuous), marital status, alcohol consumption, total energy intake (continuous), smoking status (never, former, current), diagnosis of overweight, hypertension and diabetes.

Table S3: multivariable analysis of the 5-year weight changes of participants according to weight dissatisfaction, stratified by study period and gender, CoLaus|PsyCoLaus study, Lausanne, Switzerland.

	Follow-up 1 to 2			Follow-up 2 to 3		
	**No**	**Yes**	**p-value**	**No**	**Yes**	**p-value**
**Misperception, men**						
Weight change (kg)	0.99 ± 0.17	-0.05 ± 0.37	0.011	0.11 ± 0.18	-0.73 ± 0.49	0.113
Weight change						
Loss >5 kg	-	1.66 (0.55 - 4.99)	0.370	-	9.05 (2.78 - 29.5)	<0.001
Stable	-	1 (ref)		-	1 (ref)	
Gain >5 kg	-	0.80 (0.37 - 1.75)	0.580	-	1.61 (0.48 - 5.44)	0.441
**Misperception, women**						
Weight change (kg)	0.86 ± 0.14	0.58 ± 0.19	0.246	0.22 ± 0.16	-0.28 ± 0.28	0.139
Weight change						
Loss >5 kg	-	1.93 (1.06 - 3.51)	0.031	-	1.97 (0.81 - 4.78)	0.133
Stable	-	1 (ref)		-	1 (ref)	
Gain >5 kg	-	1.20 (0.78 - 1.85)	0.408	-	1.19 (0.52 - 2.73)	0.675

Results are expressed as relative risk ratio and (95% confidence interval) for categorical variables and as adjusted mean ± standard error for continuous variables. Between-group comparisons performed using polytomous logistic regression for categorical variables and ANOVA for continuous variables. Both multivariable models were adjusted on age (continuous), marital status, alcohol consumption, total energy intake (continuous), smoking status (never, former, current), diagnosis of overweight, hypertension and diabetes.

Table S4: baseline characteristics of participants according to weight misperception or weight dissatisfaction for the 10-year study, CoLaus|PsyCoLaus study, Lausanne, Switzerland.

	Weight misperception			Weight dissatisfaction		
	**No (n=1226)**	**Yes (n=243)**	**p-value**	**No (n=1030)**	**No (n=439)**	**p-value**
Women (%)	758 (361.8)	194 (79.8)	<0.001	614 (59.6)	338 (77.0)	<0.001
Age (years)	55.5 ± 9.6	53.1 ± 8.4	<0.001	56.1 ± 9.7	52.7 ± 8.4	<0.001
Born in Switzerland (%)	829 (67.6)	175 (72.0)	0.18	714 (69.3)	290 (66.1)	0.22
Educational level (%)			0.10			<0.001
High	337 (27.5)	83 (34.2)		268 (26.0)	152 (34.6)	
Middle	364 (29.7)	69 (28.4)		301 (29.2)	132 (30.1)	
Low	525 (42.8)	91 (37.4)		461 (44.8)	155 (35.3)	
Living in couple (%)	675 (55.1)	123 (50.6)	0.20	582 (56.5)	216 (49.2)	0.010
Body mass index (kg/m^2)	22.1 ± 1.8	23.6 ± 1.2	<0.001	22.0 ± 1.9	23.2 ± 1.4	<0.001
Waist (cm)	81.1 ± 7.9	84.6 ± 7.3	<0.001	80.9 ± 7.9	83.3 ± 7.8	<0.001
Abdominal obesity (%)	65 (5.3)	51 (21.0)	<0.001	50 (4.9)	66 (15.0)	<0.001
Overweight diagnosis (%)	41 (3.4)	34 (14.4)	<0.001	35 (3.4)	40 (9.3)	<0.001
Alcohol drinker (%)	982 (80.1)	206 (84.8)	0.090	816 (79.2)	372 (84.7)	0.014
Smoking status (%)			0.66			0.76
Never	563 (45.9)	106 (43.6)		474 (46.0)	195 (44.4)	
Former	407 (33.2)	88 (36.2)		341 (33.1)	154 (35.1)	
Current	256 (20.9)	49 (20.2)		215 (20.9)	90 (20.5)	
Hypertension (%)	292 (23.8)	50 (20.6)	0.27	253 (24.6)	89 (20.3)	0.075
Diabetes (%)	29 (2.4)	1 (0.4)	§ 0.050	24 (2.3)	6 (1.4)	§ 0.24
Total energy intake (kcals)	1660 [1335-2090]	1624 [1222-1973]	† 0.019	1668 [1342-2074]	1613 [1245-2042]	† 0.047

Results are expressed as number of participants (column percentage) for categorical variables and as average ± standard or median [interquartile range] deviation for continuous variables. Between-group comparisons using chi-square or Fisher's exact test (§) for categorical variables and student's t-test or Kruskal-Wallis test (†) for continuous variables.

Table S5: bivariate and multivariable analysis of the 10-year weight changes of participants according to weight misperception or weight dissatisfaction, CoLaus|PsyCoLaus study, Lausanne, Switzerland.

	**Weight misperception**			**Weight dissatisfaction**		
	**No (n=1226)**	**Yes (n=243)**	**p-value**	**No (n=1030)**	**No (n=439)**	**p-value**
**Bivariate**						
Weight change (kg)	0.8 ± 4.5	1.6 ± 5.3	0.011	0.8 ± 4.5	1.2 ± 5.0	0.160
Weight change (%)			0.003			0.011
Loss >5 kg	76 (6.2)	19 (7.8)		59 (5.7)	36 (8.2)	
Stable	977 (79.7)	170 (70.0)		826 (80.2)	321 (73.1)	
Gain >5 kg	173 (14.1)	54 (22.2)		145 (14.1)	82 (18.7)	
**Multivariable**						
Weight change (kg)	0.87 ± 0.13	0.97 ± 0.30	0.757	0.98 ± 0.14	0.67 ± 0.22	0.245
Weight change						
Loss >5 kg	-	1.86 (1.06 - 3.26)	0.030	-	2.17 (1.36 - 3.49)	0.001
Stable	-	1 (ref)		-	1 (ref)	
Gain >5 kg	-	1.50 (1.02 - 2.19)	0.037	-	1.17 (0.85 - 1.63)	0.341
Weight change, weighted						
Loss >5 kg	-	1.71 (0.96 - 3.06)	0.069	-	2.09 (1.29 - 3.39)	0.003
Stable	-	1 (ref)		-	1 (ref)	
Gain >5 kg	-	1.52 (1.03 - 2.25)	0.036	-	1.21 (0.86 - 1.70)	0.273

For bivariate analyses, results are expressed as number of participants (column percentage) for categorical variables and as average ± standard deviation for continuous variables. Between-group comparisons using chi-square for categorical variables and student's t-test for continuous variables. For multivariable analyses, results are expressed as relative risk ratio and (95% confidence interval) for categorical variables and as adjusted mean ± standard error for continuous variables. Between-group comparisons performed using polytomous logistic regression for categorical variables and ANOVA for continuous variables. Both multivariable models were adjusted on sex, age (continuous), marital status, alcohol consumption, total energy intake (continuous), smoking status (never, former, current), diagnosis of overweight, hypertension and diabetes. Weighting using inverse probability of being included.
