# Supplementary figures and images for: Weight Misperception, Weight Dissatisfaction, and Weight Change Among a Swiss Population-Based Adult Sample
Source: Int J Environ Res Public Health. 2025 Aug 8;22(8):1237. doi: 10.3390/ijerph22081237 (PMC12386205; doi:10.3390/ijerph22081237)

## Slide 1
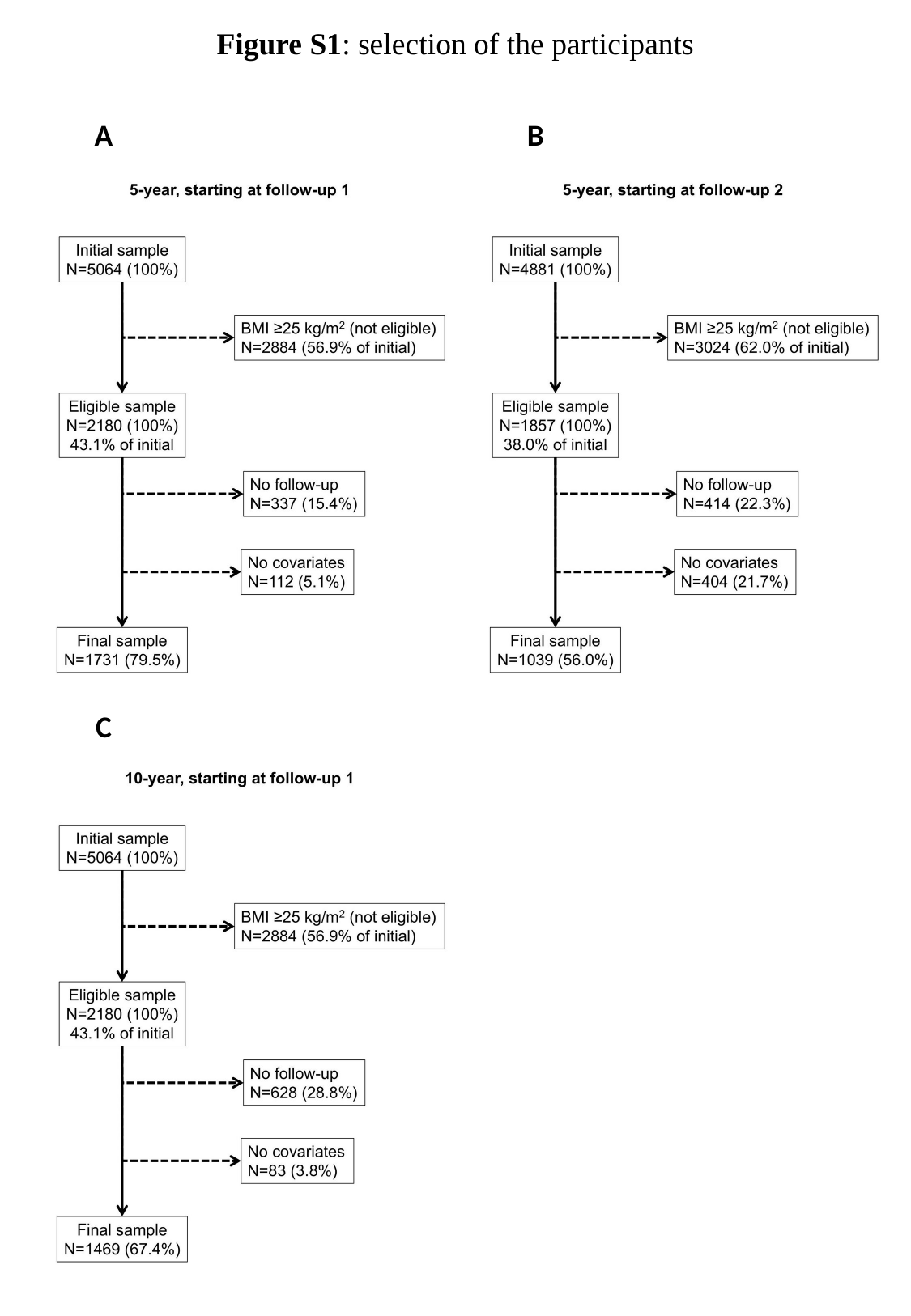

Figure S1: selection of the participants
A
B
C

Supplement: Supplementary file 1 [file ijerph-22-01237-s001.zip › Figure S1.pptx]
